# Outcomes of Directional Branches of the T-Branch Off-the-Shelf Multi-Branched Stent-Graft

**DOI:** 10.3390/jcm11216513

**Published:** 2022-11-02

**Authors:** Konstantinos Spanos, Tomasz Jakimowicz, Petroula Nana, Christian-Alexander Behrendt, Giuseppe Panuccio, George Kouvelos, Katarzyna Jama, Ahmed Eleshra, Fiona Rohlffs, Tilo Kölbel

**Affiliations:** 1 German Aortic Center Hamburg, Department of Vascular Medicine, University Heart & Vascular Center, 20251 Hamburg, Germany; 2 Department of Vascular Surgery, University Hospital of Larissa, Faculty of Medicine, School of Health Sciences, University of Thessaly, 41110 Larissa, Greece; 3 Department of General, Vascular and Transplant Surgery, Medical University of Warsaw, 02-091 Warsaw, Poland

**Keywords:** thoraco-abdominal aortic aneurysm, t-Branch, endovascular repair, off-the-shelf stent graft, bridging covered stent, balloon-expandable covered stent, self-expanding covered stent, relining

## Abstract

Background: A controversy on bridging covered stent (BCS) choice, between self-expanding (SECS) and balloon-expandable (BECS) stents, still exists in branched endovascular repair. This study aimed to determine the primary target vessel (TV) patency in patients treated with the t-Branch device and identify factors impairing the outcomes. Methods: A retrospective study was undertaken, including patients treated with the t-Branch (Cook Medical, Bloomington, IN, USA) between 2014 and 2019 (early 2014–2016; late 2017–2019). The endpoint was the primary patency (CT: celiac trunk, SMA, superior mesenteric artery, RRA: right renal artery, LRA: left renal artery) during the follow-up. Any branch instability event was assessed. The factors affecting the patency were determined using multivariable regression models and Kaplan–Meier analyses. Results: In total, 2018 TVs were analyzed; 1542 SECSs and 476 BECSs. The CT patency was 99.8% (SE 0.2%) at the 1st month, with no other event. The SMA patency was 97.8% (SE 1) at the 12th month. The RRA patency was 96.7% (SE 2) at the 24th month. The LRA patency was 99% (SE 0.4) at the 6th month. Relining was the only factor independently associated with the SMA patency (OR 8.27; 95% CI 1.4–4.9; *p* = 0.02). The freedom from instability was 62% (SE 4.3%) and 45% (SE 5.4%) at the 24th month and 36th month. No significant difference was identified between the BECSs and SECSs in the early or late experience. Conclusion: BCS for the t-Branch branches performed with a good primary patency during the short-term follow-up. The type of BCS did not influence the patency. Relining might be protective for SMA patency.

## 1. Introduction

The endovascular treatment of complex aortic aneurysms (thoracoabdominal, pararenal, and juxtarenal) has been widely accepted as a safe and effective technique [1,2,3,4,5]. Fenestrated and branched endovascular aneurysm repair (F/B-EVAR) has demonstrated high rates of technical and clinical success and low early post-operative mortality [6,7,8,9]. F/B-EVAR seems to offer a benefit regarding early mortality and perioperative complications compared to open repair [6,10]. F/B-EVAR techniques have evolved during the last two decades, thus the high-level evidence on the long-term durability of these devices is still limited, although experienced centers have reported low target vessel occlusion and reintervention rates [6,10,11].

The durability of the bridging covered stents (BCS) is of great importance in patients treated with B-EVAR using directional branches to connect to thoracoabdominal target vessels (TVs). Complications that can occur with BCSs include: endoleak, kinking, fracture, migration, occlusion, and stenosis. Over time, the combination of progression of vascular disease, endothelial proliferation, continuous arterial movement, and material fatigue may also affect TVs patency [12]. There is a controversy for the choice of BCSs for B-EVAR: a self-expanding (SECS) or balloon-expandable covered stent (BECS). The evidence is based on retrospective studies and case series, thus, currently, a high-level guideline recommendation does not exist. In general, the benefits of SECSs are their flexibility and conformability, while BECSs have a smaller profile and may offer a more precise deployment.

The aim of this study was to assess the primary patency of TVs in directional branches of patients treated with the t-Branch at two academic aortic centers, analyzing each target vessel and the factors that are associated with its patency; the secondary aim was to analyze the impact of BCSs type on its patency.

## 2. Materials and Methods

### 2.1. Study Design

The study design and early outcomes have been previously described [3]. A retrospective observational study was undertaken including two aortic centers: the German Aortic Center Hamburg, Department of Vascular Medicine, University Heart and Vascular Center, Hamburg, Germany and the Department of General, Vascular and Transplant Surgery, Medical University of Warsaw, Poland. All patients were treated using the t-Branch (Cook Medical, Bloomington, IN, USA) in an elective or urgent setting for a complex abdominal aortic aneurysm AAA [juxta-renal, supra-renal, or after previous endovascular abdominal aortic aneurysm repair (EVAR)] and thoracoabdominal aortic aneurysm (TAAA) between 2014 and 2019. The cohort was divided into two treatment periods, 2014–2016 (early) and 2017–2019 (late). No common standardized pre- or postoperative protocol existed for the two centers. The decision for B-EVAR was based on an individual patient assessment through interdisciplinary aortic boards. The data were collected at each center and then, retrospectively and anonymously inserted into a single electronic database. The demographic data, past medical history, cardiovascular risk factors, pre-operative comorbidities, intra-operative and peri-operative details, and early post-operative morbidity and mortality were also recorded according to the Strengthening the Reporting of Observational studies in Epidemiology (STROBE) statement, as it has been previously reported in detail [13]. The branch instability definition included branch stenosis or occlusion with the need of re-intervention, branch related endoleak, or dislocation [12].

A clinical evaluation and imaging using computed tomography angiography (CTA) were used for patients’ surveillance during the follow-up. This study complied with the Declaration of Helsinki. As this was a retrospective analysis of the anonymized data, no approval from the local ethics committee was required, and no patient informed consent was obtained for the study.

### 2.2. Procedure

The detailed technique of t-Branch implantation has been described previously [13]. The TVs which were attempted to be catheterized and stented were celiac trunk (CT), superior mesenteric artery (SMA), RRA (right renal artery), and LRA (left renal artery). The left axillary artery was the most used access to the directional branches. The SECSs used were Viabahn (W. L. Gore & Associates, Flagstaff, AZ, USA); Fluency (Bard Peripheral Vascular, Tempe, AZ, USA); or Covera (Bard Peripheral Vascular, Tempe, AZ, USA), while the BEGSs were Advanta V12 (Atrium Maquet Getinge Group, Mijdrecht, The Netherlands), BeGraft (Bentley, Innomed GmbH, Hechingen, Germany), or VBX (W. L. Gore & Associates, Flagstaff, AZ, USA]). Relining with a bare metal stent was at the discretion of the operator and patient’s anatomic characteristics. A relining of the bridging stents was performed using balloon-expandable or self-expanding stents (Genesis-Palmaz (Cordis Corporation, Cardinal Health company, Milpitas, CA, USA); a Zilver (Cook Medical, Bloomington, IN, USA); Protégé-EverFlex (Medtronic/Covidien, Minneapolis, MN, USA); or Wallstent (Boston Scientific Corporation, Marlborough, MA, USA)] in the case of severely angulated or tortuous visceral arteries or long distances between the visceral branch and the target vessel ostium. If a branch was not stented during the procedure, it was occluded using an Amplatzer Vascular Plug (St. Jude Medical, St. Paul, MN, USA), with or without a prior extension with a BECS, [14] and combined with a coil embolization when deemed necessary.

### 2.3. Endpoints

The endpoints were the primary patency of the bridging stents for each target vessel during the follow-up and the identification of factors that may be associated with the patency. The secondary outcomes were the impact of the BCS type, SECS vs. BECS, on the patency for each target vessel.

### 2.4. Statistical Analysis

The categorical data were expressed as the absolute numbers and/or percent prevalence (%) in the study cohort and continuous variables as the means standard deviation. In the statistical analysis for the continuous variables, the independent *t*-test for normally distributed data and the Mann–Whitney U test for nonparametric data were used. The Pearson x2 test or the Fisher exact test was used for the categorical variables, as appropriate. The primary patency of TVs and the comparison of the primary patency rates between the SECS vs. BECS with the log-rank test and Kaplan–Meier curves were generated. The univariate analysis of the factors associated with the branch patency such as one’s age, the clinical presentation, the diameter of the aneurysm, the gender, whether there was any previous aortic repair, the type of bridging covered stent (BECS vs. SECS), the number of covered stents, and the relining was undertaken for the SMA, RRA, and LRA branches. An analysis was not undertaken for CT as only one event was reported. Multivariable regression models were used to determine the independent association of the risk factors with the patency for each branch and the mortality, while controlling for possible confounders. The model selection was based on an enter procedure. The missing data was handled by KS, AE, and TJ. No correction for multiple hypothesis testing was applied. A *p* value was considered significant when it was <0.05. The statistical analysis was performed by SPSS 22.0 for Windows software (IBM Corp, Armonk, NY, USA).

## 3. Results

### 3.1. Patients’ Characteristics

Between January 2014 and December 2019, 542 patients (mean age, 70.5 ± 8.5 years; 388 males [72%]) underwent an endovascular repair using the t-Branch endograft. Based on the Crawford classification, among the patients with TAAA (90%; 487/542), 31 (5.7%) were categorized as type I, 73 (13.4%) as type II, 118 (21.7%) as type III, 233 (43%) as type IV, and 32 (5.9%) as type V. Twenty-two (4%) patients were treated due to a juxta-renal AAA (4 were symptomatic with abdominal pain, 3 presented with a contained rupture, and 15 had an AAA diameter >90 mm and it was decided by the patient not to wait for a custom-made device, that could take up to 8 weeks), 19 (3.5%) due toa supra-renal AAA and, 14 due to an AAA (contained ruptures or previously AAA intervention). Only 5 patients had a history of previous aortic dissection. The technical success rate was 97% (526/542) for the main procedure. Early outcomes have been presented recently [3]. Demographics, co-morbidities, and past medical history are shown in Table 1, distributed in two groups: patients with TV patency vs. those with a loss of patency of at least one TV during FU.

In 542 patients, 2018 TVs were connected to the directional branches of the t-Branch; 1542 TVs were connected using SECSs; and 476 using BECSs (Table 2). In 91%, 8%, and 1% of the TV, one, two, or three SECSs were used, respectively, while in 87%, 12%, and 1% of the TV, one, two, or three BECSs were used, respectively. Thus, it was more common to use >1 BCS in BECSs than in SECSs (12.6% vs. 8.7%; *p* = 0.01). A relining was performed in 86% of SECSs and 32% of BECSs (Table 2). Table 3 shows the diameters of bridging covered stents that were used.

### 3.2. Patency Rates

**Figure 1:**
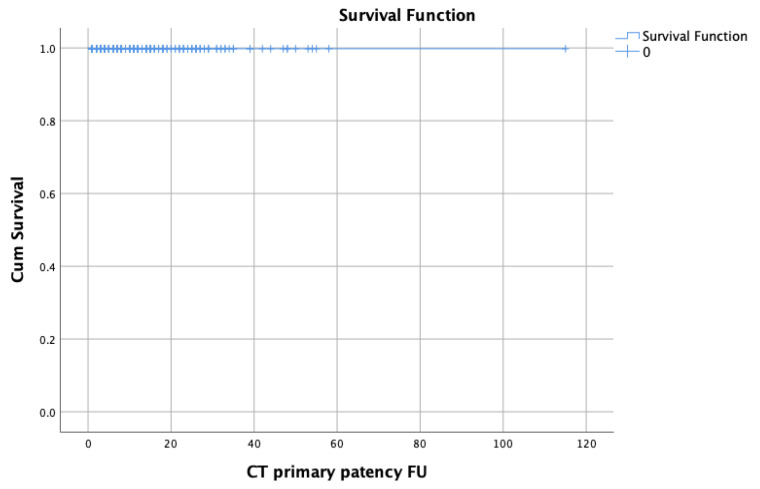

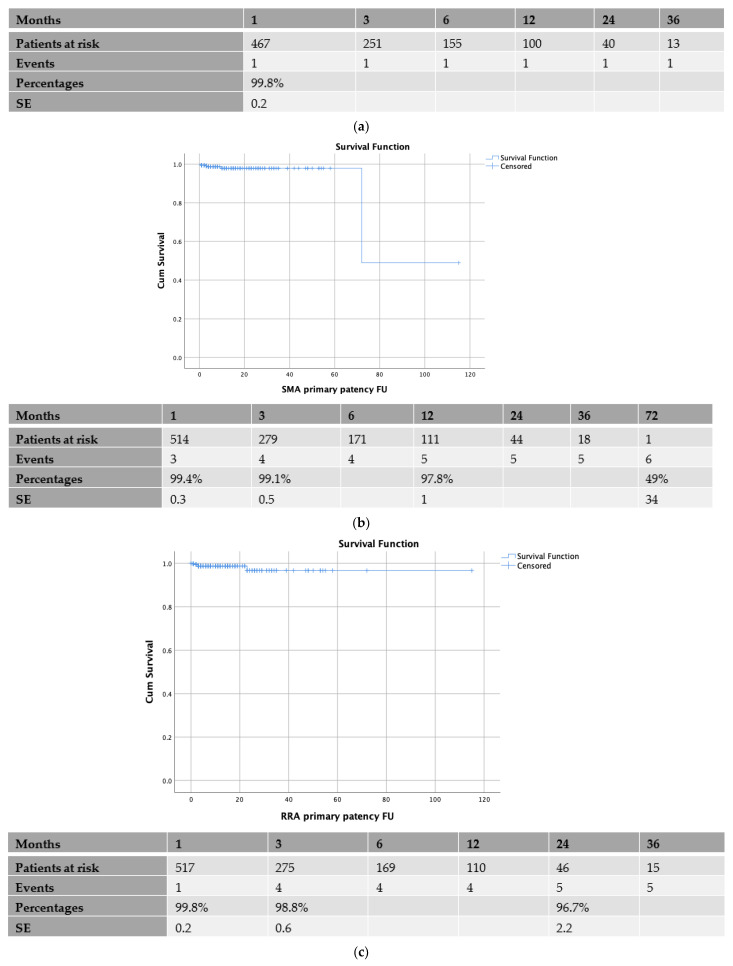

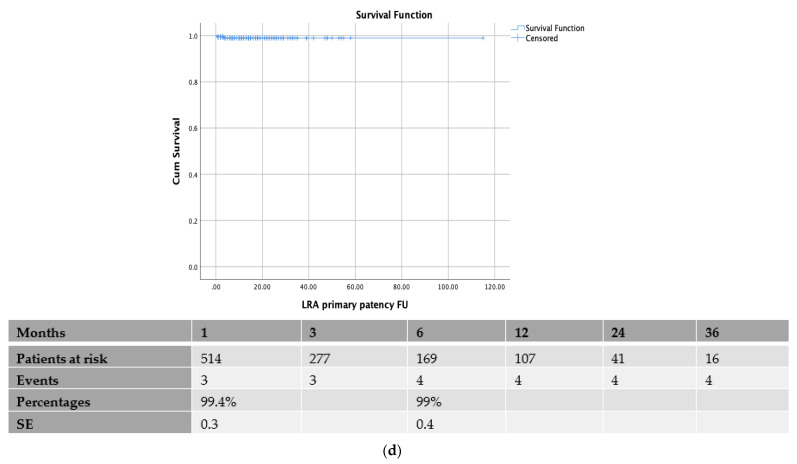
(**a**) Patency of coeliac trunk (CT) bridging covered stent. SE: standard error; (**b**) Patency of superior mesenteric artery (SMA) bridging covered stent. SE: standard error; (**c**) Patency of right renal artery (RRA) bridging covered stent. SE: standard error; (**d**) Patency of left renal artery (LRA) bridging covered stent. SE: standard error.

The CT patency rate was 99.8% (SE 0.2%) at 1 month, while no other event was identified for up to 3 years of the follow-up (Figure 1a). The SMA patency rate was 99.4% (SE 0.3%), 99.1% (SE 0.5), and 97.8% (SE 1) at the 1-, 3-, and 12-month follow-up, respectively, with no other event during the follow-up (Figure 1b). The RRA patency rate was 99.8% (SE 0.2%), 98.8% (SE 0.6), and 96.7% (SE 2) at the 1st, 3rd, and 24th month, respectively (Figure 1c). The LRA patency rate was 99.4% (SE 0.3%) and 99% (SE 0.4) at the 1st and 6th month, respectively, with no other event during the follow-up (Figure 1d). The TV patency is depicted in the Kaplan–Meier curves in Figure 1a–d.

Freedom from any TV instability was 96.8% (SE 0.8%), 93% (SE 1.3%), 88.4% (SE 1.9), 79% (SE 2.8), 62% (SE 4.3%), and 45% (SE 5.4%) at the 1st, 3rd, 6th, 12th, 24th, and 36th month (Supplementary Figure S1).

In total, twelve patients had a loss of patency in one or more TVs; in those patients, 16 TVs were occluded (Table 4). Nine patients were treated electively (9/12) and 13 of the TVs (13/16) were lost within the initial four months of the follow-up. A univariate analysis of the factors associated with the branch patency was undertaken for the SMA, RRA, and LRA branches. An analysis was not undertaken for the CT branches, as only one event was reported during the follow-up. The univariate analysis did not identify any significant factors associated with the left or right RA branch patency.

The univariate analysis for the factors affecting the SMA branch patency identified relining (with a bare metal stent) as the only associated factor. After a multivariate regression analysis (Table 5), relining with the bare metal stent was associated with a better SMA branch patency (OR 8.27; 95% CI 1.4–4.9; *p* = 0.02).

### 3.3. Balloon-Expandable vs. Self-Expanding Covered Stents

**Figure 2:**
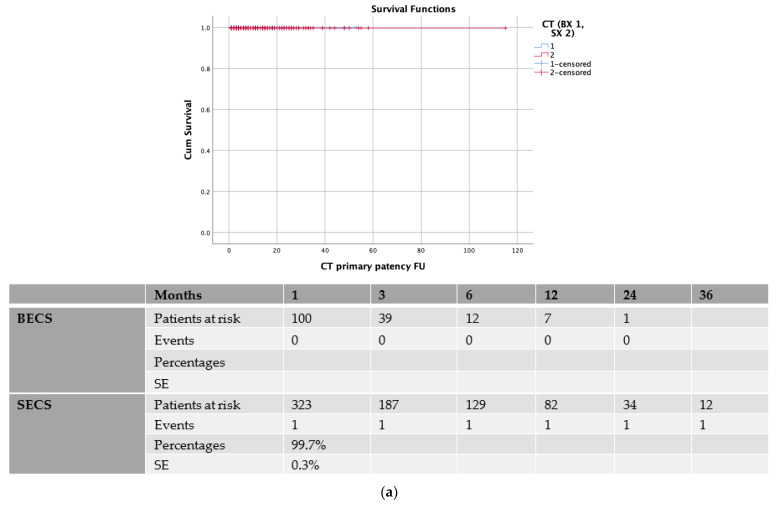

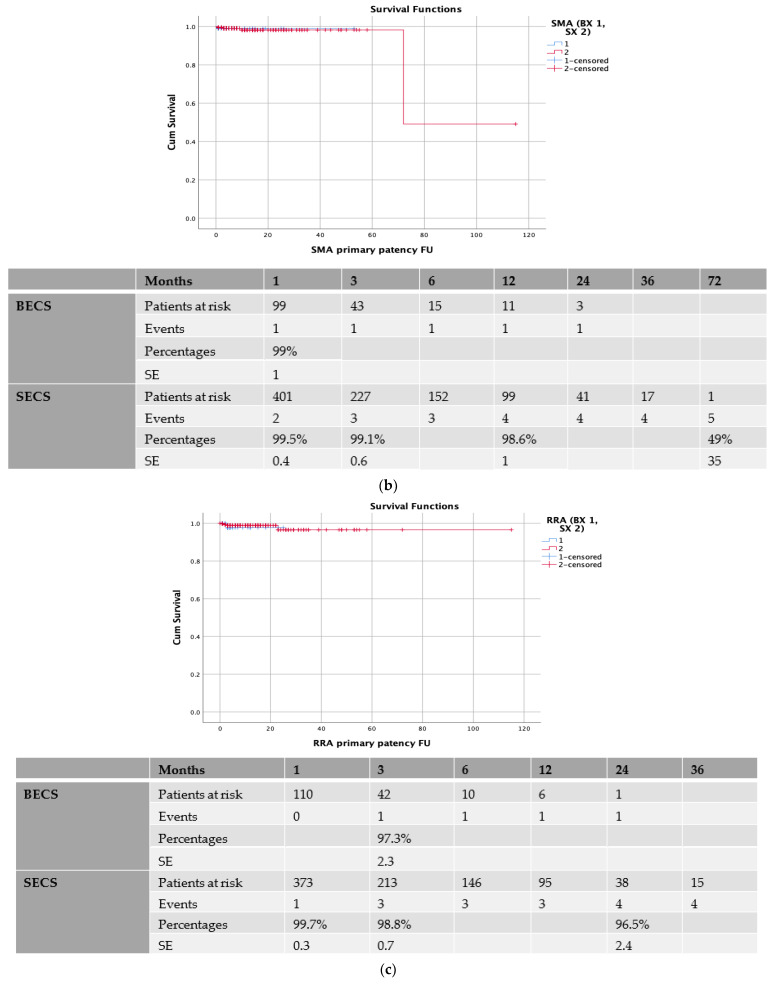

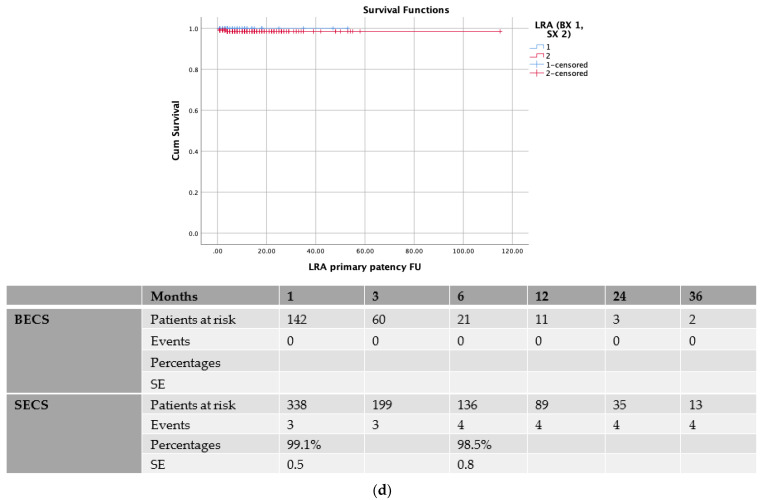
(**a**) Sub-analysis between balloon-expandable covered stents (BECSs) and self-expanding covered stents (SECSs) for primary patency of coeliac trunk (CT) bridging covered stent. SE: standard error; (**b**) Sub-analysis between balloon-expandable covered stents (BECSs) and self-expanding covered stents (SECSs) for primary patency of superior mesenteric artery (SMA) bridging covered stent. SE: standard error; (**c**) Sub-analysis between balloon-expandable covered stents (BECSs) and self-expanding covered stents (SECSs) for primary patency of right renal artery (RRA) bridging covered stent. SE: standard error; (**d**) Sub-analysis between balloon-expandable covered stents (BECSs) and self-expanding covered stents (SECSs) for primary patency of left renal artery (LRA) bridging covered stent. SE: standard error.

In the sub-analysis of the primary patency between the BECSs and SECSs, there was no statistically significant patency difference for the CT; in the BECS group, the CT patency was 100% up to 2 years, while in the SECS group, the CT patency was 99.7% (SE 0.3) at the 1st month while no other event was reported during the follow-up (*p* = 0.5, Figure 2a). There was also no difference in terms of patency for the SMA bridging stent, thus in the BECS group, the SMA patency was 99% (SE 1) at the 1st month, while no other event was reported, while in the SECS group, the SMA patency was 99.5% (SE 0.4), 99.1% (SE 0.6), and 98.6% (SE 1) at 1st, 3rd, and 12th months, respectively, with no other event during the follow-up (*p* = 0.86, Figure 2b). There was no difference in terms of the patency for the RRA bridging stent. In the BECS group, the RRA patency was 97.3% (SE 2.3) at the 3rd month, while no other event was reported, while in the SECS group, the RRA patency was 99.7% (SE 0.3), 98.8% (SE 0.7), and 96.5% (SE 2.4) at the 1st, 12th, and 24th month, respectively (*p* = 0.78, Figure 2c). Finally, no difference was noted in the patency for the LRA, thus in the BECS group, the LRA patency was 100% with no event during follow up, while in the SEGS group, the LRA patency was 99.1% (SE 0.5) and 98.5% (SE 0.8) at the 1st and 6th month, respectively, with no other event during the follow-up (*p* = 0.22, Figure 2d).

### 3.4. Early vs. Late Experience

One hundred and fifty-four patients (28%) were treated from 2014 to 2016, whereas 388 patients (72%) were treated from 2017 to 2019. In the sub-analysis in terms of the level of experience, there was no significant patency difference for the CT; in the early group, the CT patency was 99.2% (SE 0.8) in first month, with the occurrence of no other event, while in the late group, the CT patency was 100%, and no event was reported during the follow-up (*p* = 0.09, Supplementary Figure S2a). There was also no difference in terms of the patency for the SMA bridging stent, thus in the early group, the SMA patency was 98.6% (SE 0.9) and 96.8% (SE 2) at the 1st and 12th month, while in late group, the SMA patency was 99.7% (SE 0.3), 99.2% (SE 0.6), and 98.5% (SE 0.9) at the 1st, 3rd, and 6th month, respectively (*p* = 0.21), with no other event occurring (Supplementary Figure S2b). There was no difference in terms of the patency for the RRA bridging stent. In the early group, the RRA patency was 99% (SE 1) at the 3rd month, while no other event was reported, while in late group, the RRA patency was 99.7% (SE 0.3), 98.7% (SE 0.8), and 93.2% (SE 5.4) at the 1st, 12th, and 24th month, respectively (*p* = 0.39, Supplementary Figure S2c). Finally, no difference was noted in the patency for the LRA, thus in the early group, the LRA patency was 99.3% (SE 0.7) and 98% (SE 1.4) at the 1st and 6th month, with no other event occurring, while in the late group, the LRA patency was 99.5% (SE 0.4) at the 1st month, with no other event (*p* = 0.38, Supplementary Figure S2d).

## 4. Discussion

During the past decade, the endovascular treatment of complex aortic aneurysms with fenestrated and branched stent grafts became a popular option due to its low perioperative mortality and morbidity [1,2,3,4,5,6,7,8,9,15,16]. Custom-made devices (CMD), designed upon each patient’s specific anatomy, offer a dedicated treatment solution and allow an individual position of fenestrations and directional branches depending on the patient’s anatomy. However, a long production time can delay treatment and make CMDs inappropriate for the most urgent and emergency cases. The t-Branch was launched in 2012 in Europe as the first off-the-shelf standardized multibranched endograft with four directional branches for the endovascular treatment of complex aortic aneurysms, offering an alternative for patients needing either an elective or urgent management.

In a recent systematic review on the BEVAR outcomes, including seven retrospective studies and 197 patients, the primary branch patency was 98.2% during the follow-up period [17]. In the current study, which includes >500 patients who have been treated with the t-Branch, the branch patency rate was >97% at 12 months, with a low number of branch occlusion events in a follow-up of up to 3 years, confirming the good outcomes of previous studies. The level of experience did not have any impact on the outcomes. In this analysis, most of the events occurred during the initial 3 months of the follow-up. This finding highlights the need for a close surveillance in the early post-operative phase. The duplex ultrasound, along with the assessment of fenestrations and branches, may be an alternative imaging which decreases the risks associated with CTA use during the follow-up [18,19,20]. Recent studies have indicated the promising outcomes of a late revascularization of occluded renal arteries as TVs in complex endovascular aortic repair, highlighting the importance of a follow-up in those patients [21,22].

Currently, no dedicated BCS is available for use in directional branches. The available BCSs that are used are either self- or balloon-expandable and have not been designed for this purpose and their use is off-label [23]. In general, the benefits of SECSs are considered their flexibility, conformability, and longer length as the rationale for their superiority over BECSs, while BECSs have a smaller profile and may offer a more precise deployment, but >1 BCS may be needed, as it was also shown in our study [24,25,26]. In this study, we compared the use of BECS vs. SECS in terms of the patency rate in >2000 TV, by analyzing each TV separately. Both types of BCSs showed excellent outcomes in terms of patency, irrespectively the TV during follow up. To date, no RCT comparing the different covered stent types is available in the literature. Recent studies on newer balloon-expandable devices have provided conflicting outcomes; Tenorio et al. reported a significantly higher TV-instability rate for BECSs while Motta et al. reported comparable outcomes [26,27,28,29].

The relining of BCSs is used in order to reinforce compressed BCSs or smoothen a kinked distal landing zone. In this analysis, relining was more common in SECSs. The SMA branch patency was significantly better when relining was done. However, we should be cautious with this outcome, since 86% of the SECSs were relined vs. 32% of the BECSs. Additionally, it cannot be excluded that the relining of a TV could be the common practice of the physician rather than the need for a relining due to other potential reasons. No specific recommendation regarding relining has been made so far in publications and guidelines and its utilization is usually at the discretion of the operator, taking into account TVs anatomy, angulation, stenosis, or kinking [26,27,28,29]. Potentially, a combination of self-expanding and balloon-expandable stents incorporates the mechanical properties of both devices, which may have an impact on patency outcomes [25].

### Limitations

The main limitation of this study is its retrospective observational nature which introduces an unsolved challenge of residual confounding. Two experienced centers were involved, which may be adherent to different protocols for preoperative, perioperative, and postoperative practice. Another limitation is the use of bridging stents of different generations. Additionally, an analysis on different companies was not amendable since most of the times the choice of a stent-graft depended on the availability in each center. Some important information was also missing in the database, such as if there was a specific indication intra-operatively for relining. A bare metal stent for the relining was not amendable, although this would be difficult since some physicians are more aggressive with the relining while others are only so in specific cases. The availability of each bare metal stent might be also different; thus, this would be another bias. Another limitation was the absence of any analysis on the potential compression and possible kinking of the bridging stents, and the size of the aorta at the level of the renal arteries and the degree of aortic kinking at the branching site. With the use of off-the-shelf devices with a standard branch orientation, there might be in some degree a compromise with some of the branches as the positioning is not necessarily perfect for all the vessels; such an analysis was not mendable in this study. Additionally, an antiplatelet treatment could not be assessed to determine its impact on the bridging stent graft patency as there were no details on whether the patients were adherent to them during the follow-up. However, this remains the largest study presenting the primary patency outcomes of >2000 TVs.

## 5. Conclusions

The bridging covered stents for the directional branches of the t-Branch perform with good primary patency outcomes during a short-term period. The type of covered stent, either a BECS or SECS, its clinical presentation, and its previous aortic repair did not influence the patency. A relining might be protective in terms of the patency for the SMA bridging stent.

## Figures and Tables

**Table 1:** Demographics, co-morbidities, past-surgical history, and clinical presentation. CAD: coronary artery disease; HT: hypertension; HL: hyperlipidemia; COPD: chronic obstructive pulmonary disease; DM: diabetes mellitus; EVAR: endovascular aneurysm repair; AAA: abdominal aortic aneurysm; TAA: thoracic aortic aneurysm; TAAA: thoraco-abdominal aortic aneurysm.

Demographics	Total (*n* = 542)	Patency (*n* = 530)	Loss of Patency (*n* = 12)	*p* Value
Age	70.5 ± 8	71.3 ± 7	69.8 ± 11	0.77
BMI	26 ± 4	25 ± 6	23.8 ± 3	0.08
Aneurysm diameter	7.5 ± 2.5	7.6 ± 2.2	7.1 ± 1.6	0.76
Male	388 (72%)	379 (72%)	9 (75%)	0.84
Female	154 (28%)	151 (28%)	3 (25%)	
CAD	287 (53%)	284 (54%)	3 (25%)	0.028
HT	497 (92%)	485 (92%)	11 (92%)	0.35
HL	332 (61%)	329 (62%)	3 (25%)	0.004
Smoking	292 (54%)	286 (54%)	6 (50%)	0.57
COPD	102 (19%)	99 (18.6%)	3 (25%)	0.57
DM	88 (16%)	88 (16.7%)	0 (0%)	0.1
Renal insufficiency				
0	335	326 (61%)	9 (75%)	0.91
1	197	194 (37%)	3 (25%)	
2	9	9 (1.8%)	0	
3	1	1 (0.2%)	0	
Previous ascending or arch repair	25 (4.6%)	25 (5%)	0	0.42
Previous EVAR	78 (14.4%)	77 (15%)	1 (8%)	0.48
Previous AAA	90 (16.6%)	85 (16%)	5 (42%)	0.033
Previous TAA	72 (13.3%)	68 (13%)	4 (33%)	0.06
Status of aneurysm				
Non-ruptured	339	330 (62%)	9 (75%)	0.46
Contained	46	45 (8.5%)	1 (8%)	
Symptomatic	157	155 (29%)	2 (16%)	
Type of aneurysm				
Juxta-renal	22	22	0	
Supra-renal	19	19	0	
TAAA Type I	31	31	0	
TAAA Type II	73	70	3	
TAAA Type III	118	117	1	
TAAA Type IV	233	228	5	
TAAA Type V	32	30	2	
Infra-renal	14	13	1	

**Table 2:** This table shows type and number of bridging covered stents that were used for each target vessel. CT: coeliac trunk; SMA: superior mesenteric artery; RRA: right renal artery; LRA: left renal artery; BX: balloon expandable; SX: self-expanding.

	Total	BX	1	2	3	Relining	1	2	SX	1	2	3	Relining	1	2	3
**CT**	485	110	97	13	0	30 (27%)	30	0	375	356	17	2	295 (79%)	290	5	0
**SMA**	520	101	86	14	1	27 (27%)	27	0	419	394	21	4	342 (82%)	337	5	0
**RRA**	506	116	107	8	1	42 (36%)	40	2	390	344	45	1	305 (78%)	291	13	1
**LRA**	507	149	126	23	0	54 (36%)	52	2	358	314	41	3	276 (77%)	254	11	1
**Total**	2018	476	416	58	2	153 (32%)	149	4	1542	1408	124	10	1218 (86%)	1172	34	2

**Table 3:** This table shows the diameters of bridging covered stents that were used. BX: balloon expandable; SX: self-expanding; CT: coeliac trunk; SMA: superior mesenteric artery; RRA: right renal artery; LRA: left renal artery.

	Total	CT			SMA			RRA			LRA		
mm		N	BX	SX	N	BX	SX	N	BX	SX	N	BX	SX
5	2	0	0	0	0	0	0	0	0	0	2	2	0
6	500	5	3	2	3	2	1	254	55	199	238	67	171
7	239	6	3	3	10	2	8	108	17	91	115	24	91
8	537	230	48	182	210	37	173	51	6	45	46	14	32
9	201	80	20	60	106	23	83	2	0	2	13	4	9
10	157	65	10	55	87	14	73	2	1	1	3	1	2
11	2	2	0	2	0	0	0	0	0	0	0	0	0
12	26	11	0	11	15	0	15	0	0	0	0	0	0

**Table 4:** Description of patients with loss of patency in one or more target vessels. AAA: abdominal aortic aneurysm; TAAA: thoraco-abdominal aortic aneurysm; BCS: bridging covered stents; BECS: balloon-expandable covered stent; SECS: self-expanding covered stent.

N	Age	AAA Diameter	Clinical Presentation	Type of Aneurysm	TV Occlusion	Time of Event in Months	Type of BCS	Number of BCS	Relining	Number of BMS
1	74	7.4	Asymptomatic	**Type IV TAAA**	RRA	2	SECS	1	1	1
2	85	6.2	Asymptomatic	**Type III TAAA**	LRA	1	SECS	1	1	1
					SMA	1	SECS	1	1	1
					CT	1	SECS	1	0	0
3	79	7	Asymptomatic	**Type IV TAAA**	SMA	1	SECS	1	1	1
4	66	6.8	Asymptomatic	**Type IV TAAA**	RRA	3	BECS	1	1	1
5	82	6.7	Contained ruptured	**Type V TAAA**	SMA	1	BECS	1	0	0
6	44	12	Asymptomatic	**Type IV TAAA**	LRA	1	SECS	1	1	1
7	68	6.5	Asymptomatic	**Type IV TAAA**	RRA	3	SECS	1	1	1
8	66	5.7	Asymptomatic	**Type II TAAA**	LRA	4	SECS	1	0	0
					SMA	72	SECS	1	0	0
9	72	6.3	Symptomatic	**Type II TAAA**	SMA	10	SECS	2	0	0
10	61	5.8	Asymptomatic	**Infrarenal**	RRA	23	SECS	1	0	0
11	57	7.1	Asymptomatic	**Type V TAAA**	SMA	3	SECS	1	0	0
12	78	6.5	Symptomatic	**Type II TAAA**	RRA	1	SECS	1	0	0
					LRA	1	SECS	1	0	0

**Table 5:** Univariate and multivariate analysis of factors associated with target vessel patency. SMA: superior mesenteric artery; RRA: right renal artery; LRA: left renal artery; CT: coeliac trunk; BX: balloon expandable; SX: self-expanding.

Univariate Analysis of SMA Patency		Multivariate			CI	
Age	0.28	Relining	0.02	8.27	1.4	49
Clinical presentation	0.36	BX vs. SX	0.26			
Diameter of aneurysm	0.72					
Gender	0.23					
Previous aortic repair	0.65					
BX vs. SX	0.11					
Relining	0.01					
Numbers of stents	0.83					
**Univariate analysis of RRA patency**						
Age	0.89					
Clinical presentation	0.44					
Diameter of aneurysm	0.71					
Gender	0.76					
Previous aortic repair	0.17					
BX vs. SX	0.48					
Relining	0.42					
Numbers of stents	0.21					
**Univariate analysis of LRA patency**						
Age	0.62					
Clinical presentation	0.36					
Diameter of aneurysm	0.81					
Gender	0.35					
Previous aortic repair	0.67					
BX vs. SX	0.07					
Relining	0.14					
Numbers of stents	0.3					
**Univariate analysis of LRA patency**	Not feasible with only one event					

## Data Availability

The data presented in this study are available on request from the corresponding author.

## Supplementary Materials

The following supporting information can be downloaded at: https://www.mdpi.com/article/10.3390/jcm11216513/s1, Figure S1: freedom from target vessel instability. SE: standard error; Figure S2: (a) sub-analysis between early and late experience for primary patency of coeliac trunk (CT) bridging covered stent. SE: standard error; (b) sub-analysis between early and late experience for primary patency of superior mesenteric artery (SMA) bridging covered stent. SE: standard error; (c) sub-analysis between early and late experience for primary patency of right renal artery (RRA) bridging covered stent. SE: standard error; (d) sub-analysis between early and late experience for primary patency of left renal artery (LRA) bridging covered stent. SE: standard error.

## References

[1] Spanos K., Kölbel T., Kubitz J.C., Wipper S., Konstantinou N., Heidemann F., Rohlffs F., Debus S.E., Tsilimparis N. (2019). Risk of spinal cord ischemia after fenestrated or branched endovascular repair of complex aortic aneurysms. J. Vasc. Surg..

[2] Makaloski V., Tsilimparis N., Panuccio G., Spanos K., Wyss T.R., Rohlffs F., Debus E.S., Kölbel T. (2021). Perioperative Outcome of Fenestrated and Branched Stent Grafting after Previous Open or Endovascular Abdominal Aortic Repair. Ann. Vasc. Surg..

[3] Kölbel T., Spanos K., Jama K., Behrendt C.-A., Panuccio G., Eleshra A., Rohlffs F., Jakimowicz T. (2021). Early outcomes of the t-Branch off-the-shelf multi-branched stent graft in 542 patients for elective and urgent aortic pathologies: A retrospective observational study. J. Vasc. Surg..

[4] Eleshra A., Hatm M., Spanos K., Panuccio G., Rohlffs F., Debus E.S., Behrendt C.-A., Tsilimparis N., Kölbel T. (2021). Early outcomes of t-Branch off-the-shelf multibranched stent-graft in urgent and emergent repair of thoracoabdominal aortic aneurysms. J. Vasc. Surg..

[5] Spanos K., Kölbel T., Theodorakopoulou M., Heidemann F., Rohlffs F., Debus E.S., Tsilimparis N. (2018). Early Outcomes of the t-Branch Off-the-Shelf Multibranched Stent-Graft in Urgent Thoracoabdominal Aortic Aneurysm Repair. J. Endovasc. Ther..

[6] Mastracci T.M., Eagleton M.J., Kuramochi Y., Bathurst S., Wolski K. (2015). Twelve-year results of fenestrated endografts for juxtarenal and group iv thoracoabdominal aneurysms. J. Vasc. Surg..

[7] Oderich G.S., Ribeiro M., Hofer J., Wigham J., Cha S., Chini J., Macedo T.A., Gloviczki P. (2017). Prospective, nonrandomized study to evaluate endovascular repair of pararenal and thoracoabdominal aortic aneurysms using fenestrated-branched endografts based on supraceliac sealing zones. J. Vasc. Surg..

[8] Walker J., Kaushik S., Hoffman M., Gasper W., Hiramoto J., Reilly L., Chuter T. (2019). Long-term durability of multibranched endovascular repair of thoracoabdominal and pararenal aortic aneurysms. J. Vasc. Surg..

[9] Spanos K., Antoniou G.A., Giannoukas A.D., Rohlffs F., Tsilimparis N., Debus S.E., Koelbel T. (2018). Durability of fenestrated endovascular aortic repair for juxta-renal abdominal aortic aneurysm repair. J. Cardiovasc. Surg..

[10] Oderich G.S., Farber M.A., Schneider D., Makaroun M., Sanchez L.A., Schanzer A., Beck A.W., Starnes B.W., Fillinger M., Tenorio E.R. (2021). Final 5-year results of the United States Zenith Fenestrated prospective multicenter study for juxtarenal abdominal aortic aneurysms. J. Vasc. Surg..

[11] Verhoeven E.L.G., Katsargyris A., Bekkema F., Oikonomou K., Zeebregts C.J.A.M., Ritter W., Tielliu I.F.J. (2015). Editor’s choice—ten-year experience with endovascular repair of thoracoabdominal aortic aneurysms: Results from 166 consecutive patients. Eur. J. Vasc. Endovasc. Surg..

[12] Mastracci T.M., Greenberg R.K., Eagleton M.J., Hernandez A.V. (2013). Durability of branches in branched and fenestrated endografts. J. Vasc. Surg..

[13] Tsilimparis N., Fiorucci B., Debus E.S., Rohlffs F., Kölbel T. (2017). Technical aspects of implanting the t-Branch off-the-shelf multibranched stentgraft for thoracoabdominal aneurysms. J. Endovasc. Ther..

[14] Tenorio E.R., Oderich G.S., Kölbel T., Gargiulo M., Timaran C.H., Bertoglio L., Modarai B., Jama K., Eleshra A., Lima G.B. (2021). Trans-Atlantic Aortic Research Consortium. Outcomes of off-the-shelf multi-branched stent grafts with intentional occlusion of directional branches using endovascular plugs during endovascular repair of complex aortic aneurysms. J. Vasc. Surg..

[15] Eleshra A., Oderich G.S., Spanos K., Panuccio G., Kärkkäinen J.M., Tenorio E.R., Kölbel T. (2020). Short-term outcomes of the t-Branch off-the-shelf multibranched stent graft for reintervention after previous infrarenal aortic repair. J. Vasc. Surg..

[16] Bosiers M., Kölbel T., Resch T., Tsilimparis N., Torsello G., Austermann M. (2021). Early and mid-term results from a postmarket observational study of Zeenith t-Branch thoracoabdominal endovascular graft. J. Vasc. Surg..

[17] Konstantinou N., Antonopoulos C.N., Jerkku T., Banafsche R., Kölbel T., Fiorucci B., Tsilimparis N. (2020). Systematic review and meta-analysis of published studies on endovascular repair of thoracoabdominal aortic aneurysms with the t-Branch off-the-shelf multibranched endograft. J. Vasc. Surg..

[18] Zierler R.E. (2020). Duplex ultrasound follow-up after fenestrated and branched endovascular aneurysm repair (FEVAR and BEVAR). Semin. Vasc. Surg..

[19] Perini P., Sediri I., Midulla M., Delsart P., Gautier C., Haulon S. (2012). Contrast-enhanced ultrasound vs. CT angiography in fenestrated EVAR surveillance: A single-center comparison. J. Endovasc. Ther..

[20] Gargiulo M., Gallitto E., Serra C., Freyrie A., Mascoli C., Massoni C.B., De Matteis M., De Molo C., Stella A. (2014). Could four-dimensional contrast-enhanced ultrasound replace computed tomography angiography during follow up of fenestrated endografts? Results of a preliminary experience. Eur. J. Vasc. Endovasc. Surg..

[21] Konstantinou N., Kölbel T., Dias N.V., Verhoeven E., Wanhainen A., Gargiulo M., Oikonomou K., Verzini F., Heidemann F., Sonesson B. (2021). Revascularization of occluded renal artery stent grafts after complex endovascular aortic repair and its impact on renal function. J. Vasc. Surg..

[22] Heidemann F., Kölbel T., Debus E.S., Diener H., Carpenter S.W., Rohlffs F., Tsilimparis N. (2018). Renal Function Salvage After Delayed Endovascular Revascularization of Acute Renal Artery Occlusion in Patients with Fenestrated-Branched Endovascular Aneurysm Repair or Visceral Debranching. J. Endovasc. Ther..

[23] Bertoglio L., Loschi D., Cambiaghi T., Mascia D., Kahlberg A.L., Melissano G., Chiesa R. (2018). Preliminary Outcomes of the Lifestream Balloon-Expandable Covered Stent in Fenestrated and Branched Thoracoabdominal Endovascular Repairs. J. Endovasc. Ther..

[24] Piazza M., Squizzato F., Xodo A., Gubert A., Grego F., Antonello M. (2021). Effect of branch length and tortuosity on the outcomes of branched endovascular repair of thoracoabdominal aneurysms using self-expandable bridging stent-graft. J. Vasc. Surg..

[25] Mastracci T., Carrell T., Constantinou J., Dias N., Martin-Gonzalez T., Katsargyris A., Modarai B., Resch T., Verhoeven E., Burnell M. (2016). Editor’s Choice-Effect of Branch Stent Choice on Branch-related Outcomes in Complex Aortic Repair. Eur. J. Vasc. Endovasc. Surg..

[26] Tenorio E.R., Kärkkäinen J.M., Mendes B.C., DeMartino R.R., Macedo T.A., Diderrich A., Hofer J., Oderich G.S. (2020). Outcomes of directional branches using self-expandable or balloon-expandable stent grafts during endovascular repair of thoracoabdominal aortic aneurysms. J. Vasc. Surg..

[27] Abisi S., Gkoutzios P., Carmichael M., Patel S., Sallam M., Donati T., Zayed H. (2021). The Early Outcomes of BeGraft Peripheral Plus in Branched Endovascular repair of Thoracoabdominal Aneurysms. J. Endovasc. Ther..

[28] Gallitto E., Faggioli G., Fenelli C., Mascoli C., Pini R., Ancetti S., Logiacco A., Sonetto A., Gargiulo M. (2020). The combined Use of Distal Self-Expandable and Proximal Balloon Expandable Stent Graft in Bridging Hostile Renal Arteries in Thoracoabdominal Branched Endografting. Ann. Vasc. Surg..

[29] Motta F., Parodi F.E., Knowles M., Crowner J.R., Pascarella L., McGinigle K.L., Marston W.A., Kibbe M.R., Ohana E., Farber M.A. (2021). Performance of Viabahn balloon-expandable stent compared with self-expandable covered stents for branched endovascular aortic repair. J. Vasc. Surg..

