# Supplementary material for: Outcomes of Directional Branches of the T-Branch Off-the-Shelf Multi-Branched Stent-Graft
Source: J Clin Med. 2022 Nov 2;11(21):6513. doi: 10.3390/jcm11216513 (PMC9659142; doi:10.3390/jcm11216513)
Supplement: Supplementary file 1 [file jcm-11-06513-s001.zip › jcm-1937692-supplementary.pdf]

Supplementary Figure S1. Freedom from target vessel instability. SE: standard error.

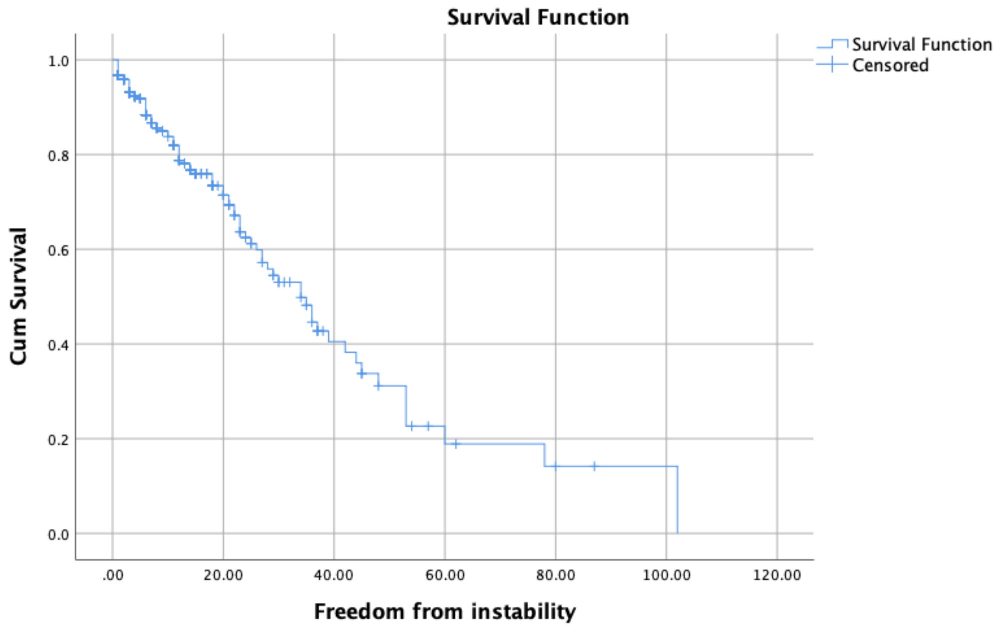


| Months | 1 | 3 | 6 | 12 | 24 | 36 |
|---|---|---|---|---|---|---|
| Patients at risk | 508 | 274 | 180 | 124 | 52 | 25 |
| Events | 17 | 28 | 38 | 54 | 71 | 83 |
| Percentages | 96.8% | 93% | 88.4% | 79% | 62% | 45% |
| SE | 0.8 | 1.3 | 1.9 | 2.8 | 4.3 | 5.4 |

Supplementary Figure S2. (**a**). Sub-analysis between early and late experience for primary patency of coeliac trunk (CT) bridging covered stent. SE: standard error. (**b**). Sub-analysis between early and late experience for primary patency of superior mesenteric artery (SMA) bridging covered stent. SE: standard error. (**c**). Sub-analysis between early and late experience for primary patency of right renal artery (RRA) bridging covered stent. SE: standard error. (**d**). Sub-analysis between early and late experience for primary patency of left renal artery (LRA) bridging covered stent. SE: standard error.

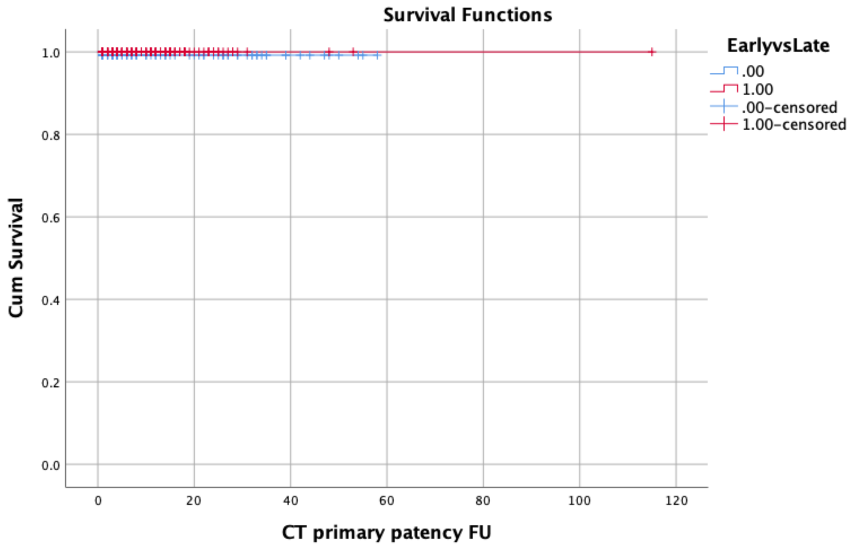


(p=0.09)

| Months | 1 | 3 | 6 | 12 | 24 | 36 |
|---|---|---|---|---|---|---|
| **Patients at risk Early** | 125 | 80 | 57 | 41 | 21 | 12 |
| **Events** | 1 | 1 | 1 | 1 | 1 | 1 |
| **Percentages** | 99.2 | | | | | |
| **SE** | 0.8 | | | | | |
| **Patients at risk Early** | 341 | 170 | 97 | 58 | 13 | 3 |

| | | | | | | |
|---|---|---|---|---|---|---|
| **Events** | 0 | 0 | 0 | 0 | 0 | 0 |
| **Percentages** | | | | | | |
| **SE** | | | | | | |

(**a**)

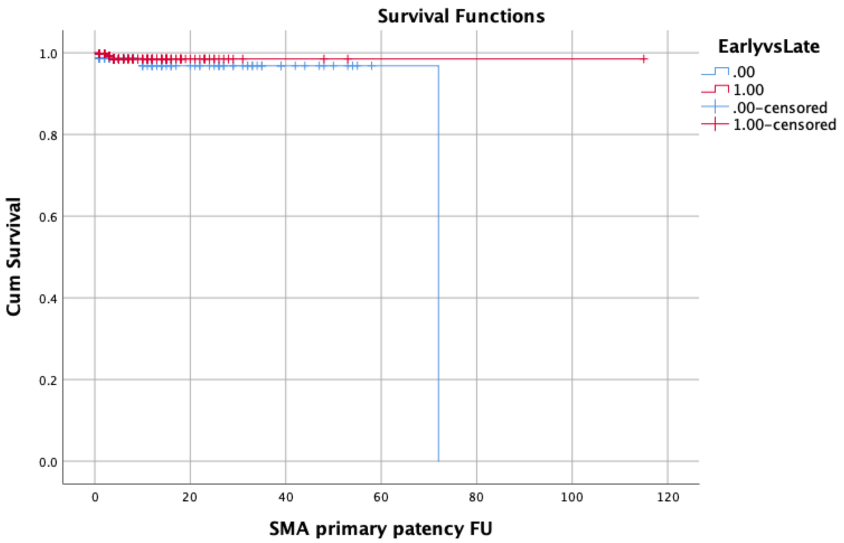


(p=0.21)

| **Months** | **1** | **3** | **6** | **12** | **24** | **36** |
|---|---|---|---|---|---|---|
| **Patients at risk Early** | 146 | 92 | 66 | 48 | 30 | 13 |
| **Events** | 2 | 2 | 2 | 3 | 3 | 3 |
| **Percentages** | 98.6% | | | 96.8 | | |
| **SE** | 0.9 | | | 2 | | |
| **Patients at risk Early** | 368 | 186 | 104 | 62 | | |
| **Events** | 1 | 2 | 3 | 3 | 13 | 3 |
| **Percentages** | 99.7 | 99.2 | 98.5 | | 3 | 3 |
| **SE** | 0.3 | 0.6 | 0.9 | | | |

(**b**)

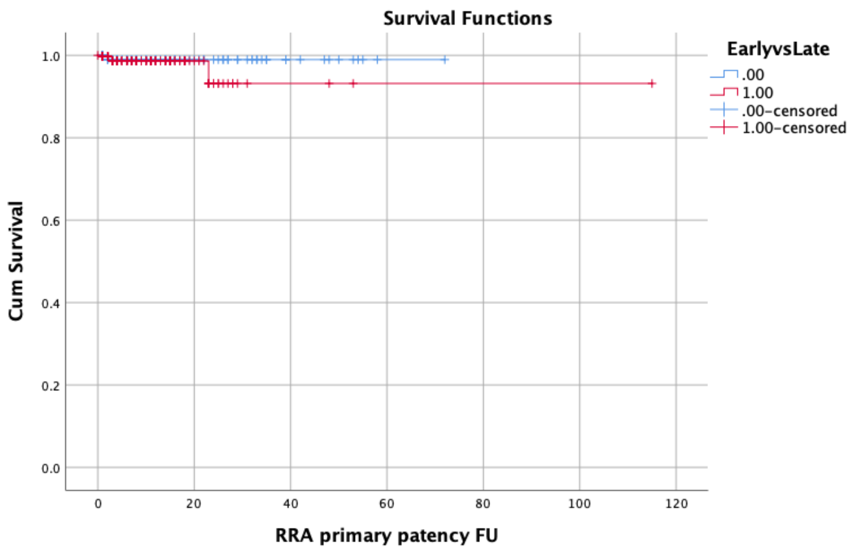


(p=0.39)

| Months | 1 | 3 | 6 | 12 | 24 | 36 |
|---|---|---|---|---|---|---|
| Patients at risk Early | 144 | 91 | 65 | 48 | 28 | 12 |
| Events | 0 | 1 | 1 | 1 | 1 | 1 |
| Percentages | | 99 | | | | |
| SE | | 1 | | | | |
| Patients at risk Early | 372 | 183 | 103 | 61 | 12 | |
| Events | 1 | 3 | 3 | 3 | 4 | 4 |
| Percentages | 99.7 | 98.7 | | | 93.2 | 3 |
| SE | 0.3 | 0.8 | | | 5.4 | |

(c)

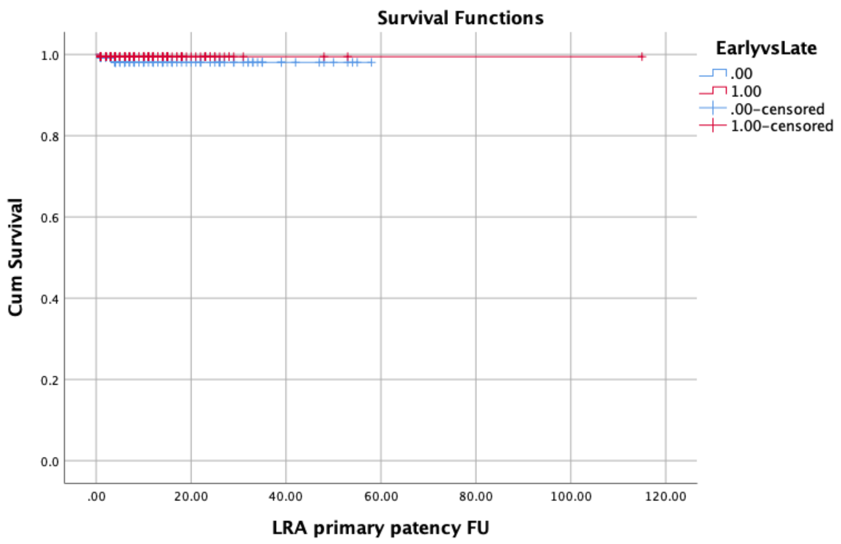


(p=0.38)

| Months | 1 | 3 | 6 | 12 | 24 | 36 |
|---|---|---|---|---|---|---|
| **Patients at risk Early** | 146 | 92 | 64 | 47 | 27 | 11 |
| **Events** | 1 | 1 | 2 | 2 | 2 | 1 |
| **Percentages** | 99.3 | | 98 | | | |
| **SE** | 0.7 | | 1.4 | | | |
| **Patients at risk Early** | 368 | 184 | 104 | 59 | 13 | 3 |
| **Events** | 2 | 2 | 2 | 2 | 2 | 2 |
| **Percentages** | 99.5 | | | | | |
| **SE** | 0.4 | | | | | |

**(d)**
